# Finding connections in the unexpected detection of *Plasmodium vivax* and *Plasmodium falciparum* DNA in asymptomatic blood donors: a fact in the Atlantic Forest

**DOI:** 10.1186/1475-2875-13-337

**Published:** 2014-08-28

**Authors:** Maria Anice Mureb Sallum, Cláudio Tadeu Daniel-Ribeiro, Gabriel Zorello Laporta, Maria de Fátima Ferreira-da-Cruz, Luciana Morganti Ferreira Maselli, Débora Levy, Sérgio Paulo Bydlowski

**Affiliations:** Departamento de Epidemiologia, Faculdade de Saúde Pública, Universidade de São Paulo, São Paulo, SP 01246-904 Brazil; Laboratório de Pesquisa em Malária, Instituto Oswaldo Cruz, Fiocruz, Rio de Janeiro, RJ 21045-900 Brazil; Fundação Pró-Sangue Hemocentro de São Paulo, Faculdade de Medicina da Universidade de São Paulo, São Paulo, SP 05403-000 Brazil; Laboratório de Genética e Hematologia Molecular (LIM31), Faculdade de Medicina da Universidade de São Paulo, São Paulo, SP 05403-000 Brazil

**Keywords:** Malaria, *Plasmodium falciparum*, *Plasmodium vivax*, Subclinical infection, Blood donors, Atlantic Forest

## Abstract

A recent paper in *Malaria Journal* reported the observation of unexpected prevalence rates of healthy individuals carrying *Plasmodium falciparum* (5.14%) or *Plasmodium vivax* (2.26%) DNA among blood donors from the main transfusion centre in the metropolitan São Paulo, a non-endemic area for malaria. The article has been challenged by a group of authors who argued that the percentages reported were higher than those found in blood banks of the endemic Amazon Region and also that that paper had not considered the literature on the classical dynamics of malaria transmission in the Atlantic Forest, which involves *Anopheles* (*Kerteszia*) *cruzii* and bromeliad malaria, due to *P. vivax* and *Plasmodium malariae* parasites, but not *P. falciparum*. The present commentary paper responds to this challenge and brings evidence and literature data supporting that the observed prevalence ratios may indicate a proportion of individuals that are exposed to *Plasmodium* transmission in permissive environments; that blood carrying parasite DNA may not be necessarily infective if used in transfusion; and that in the literature, there are examples supporting the circulation of *P. falciparum* in the area.

## Background

The dynamics of malaria in the Brazilian Atlantic Forest seems to represent ideal, low transmission settings that involve patterns of evolution of *Plasmodium* parasites, its hosts and its vectors. Evidence of subclinical plasmodial infection in inhabitants and visitors of forest areas indicate that the dynamics of malaria may have reached a sustainable transmission level, without causing morbidity and affecting the survival of hosts, reservoirs and vectors. Following the intensive and successful malaria-eliminating programme from the 1950s onwards, *Plasmodium falciparum* and *Plasmodium vivax* reservoirs and mosquito vectors may have remained clustered in small geographical areas that maintain the circulation of the *Laverania* protozoan without the costs of malaria burden. This ideal evolution scenery needs further investigations in order to find connections in the unexpected high prevalence ratio of asymptomatic infection in humans exposed to forest environments. The study by Maselli *et al.* [1] points to the maintenance of *P. falciparum* in the Atlantic Forest in silent cycles that may involve non-human primates, humans and mosquito vectors with distinct biology and ecological features, beyond the classical *Kerteszia*/bromeliad malaria transmission dynamics.

Fully understanding malaria transmission represents a challenge for the disease control and the maintenance of the effectiveness of interventions. In countries where malaria was successfully eliminated, the epidemiology of the disease becomes more complex [2]. It is, therefore, possible to assume that after an exhaustive, successful control, the residual malaria in the Atlantic Forest may encompass distinct dynamics of transmission. In this biome, the epidemiology of malaria seems to be determined by biologically distinct vectors, wild non-human primates and humans, in a conducive, heterogeneous environment.

Among more than 400 anophelines, approximately 70 species [3] can transmit the six Apicomplexan protozoan of the genus *Plasmodium* that can infect and cause malaria in humans, *P. falciparum*, *P. vivax*, *Plasmodium knowlesi*, *Plasmodium malariae*, *Plasmodium ovale curtisi* and *Plasmodium ovale wallikeri* [4]. Sinka [3] clearly shows that in Brazil, in addition to *Anopheles cruzii* other species of the subgenera *Anopheles*, *Kerteszia* and *Nyssorhynchus* are potential vectors of *Plasmodium* protozoans. Considering that members of the three subgenera are biologically and ecologically distinct, the dynamics of malaria in Brazilian ecosystem seems to encompass complex dynamics.

According to Begon [5], a parasite is an “organism that obtains its nutrients from one or a very few hosts”, whereas a pathogen is “any parasite that gives rises to a disease”. Additionally, Begon argues that both a parasite and a host population have their own interacting dynamics. Consequently, the knowledge of epidemiology of malaria may address the determinants associated to humans, mosquitoes, *Plasmodium* protozoan (that can behave either as parasite or pathogen) and other potential host/reservoir populations.

The origin and evolutionary history of *P. falciparum* has been subject of recent studies and a seminal work on *Plasmodium* changed the paradox of the origin of the human *P. falciparum.* By testing faeces samples from wild-living apes throughout central Africa, Liu *et al.* [6] showed that *Plasmodium* infections were highly prevalent and widely distributed in the region. Moreover, western gorillas (*Gorilla gorilla*) were infected with a *Plasmodium* nearly identical to *P. falciparum*. Results of phylogenetic analyses using mitochondrial genome sequences showed that human *P. falciparum* clustered within gorilla *P. falciparum* lineage, indicating that the parasite is of gorilla origin. Rayner *et al.* [7] proposed the name G1 for the phylogenetic lineage that included *Plasmodium* parasites from the subgenus *Laverania*, i.e., human *P. falciparum* and *P. praefalciparum* for the parasites from gorillas as precursor of human *P. falciparum*. Based on evidence that the gorilla *P. falciparum* has been transferred to humans, it is possible that additional cross-species transfer has occurred in other regions where non-human primates live in close association with other potential reservoirs. Garamszegi [8] suggested that parasites may preferentially infect hosts that provide adequate conditions for their reproduction. Furthermore, Sharp *et al.* [9] predicted that the emergence of *P. falciparum* was likely caused by changes in human demography and behaviour that opened new niches that cause the emergence of the parasite in humans. In agreement, Silva *et al.* [10] suggested that ape infections with *P. falciparum*-like parasites are a consequence of deforestation and land use. These intensive ecological changes brought humans in close contact with wild-living apes, facilitating the transmission of parasites among infected apes and susceptible humans, and the other way around.

In Brazil, the Atlantic Forest biome has undergone massive and intensive ecological changes since the beginning of European colonization in the Sixteen Century [11]. Currently, it is estimated that only 11.4–16.0% of the original forest cover remains, most as fragments [12]. As a consequence of the fragmentation, humans and wild non-human primates may be in contact. This situation seems to occur in forest-fragmented regions of the Atlantic Forest, for instance in Juquitiba municipality, in areas of the northeastern coast of the São Paulo state, and in the southern of the São Paulo municipality. In the Parelheiros subdistrict of the São Paulo municipality, there are small fragments of Atlantic Forest, where autochthonous *P. vivax* malaria were notified (see [13]) and a few mosquito species other than *An. cruzii* were tested positive for the parasite [14].

In cross sectional study by Maselli *et al.* [1], the blood donors that had plasmodial infection either lived in areas of the east region of São Paulo state or had visited localities situated on the coast where most of the forest fragment remains. In striking contrast with the classical bromeliad-malaria model [15], most of the subclinical infections were associated with *P. falciparum*. Likely, the positivity observed was a consequence of a single template TaqMan-based real-time PCR with specific probes for each *Plasmodium* species adopted as the standard protocol for testing blood samples. The single template real time PCR that tested positive for *P. falciparum* (prevalence of *Pf* = 5.14) of the blood donors is indicating that a *P. falciparum* or *P. falciparum*-like parasite is infecting humans without causing disease. Three evidences corroborate this hypothesis. The first one is a single case of *P. falciparum* malaria among 14 autochthonous malarious patients from areas of the Atlantic Forest that were diagnosed and treated at the Instituto Nacional de Infectologia Evandro Chagas in Fiocruz (from 2008 to 2013). All 14 patients had acute vivax malaria, and a single patient was found infected by both *P. falciparum* and *P. vivax*. This makes possible that the *P. falciparum* infection was detected coincidentally only as a consequence of the concomitant (symptom-inducing) *P. vivax* infection (Pina-Costa, Doctoral Thesis, Instituto Nacional de Infectologia, Fiocruz, August 2014). The second evidence is provided by a recent publication by Abkallo *et al.* [16], showing that *Plasmodium* DNA from a pre-erythrocytic stage can be detected both in blood and faeces from infected non-human primates in the absence of blood stage parasites. This event, indicating that not all infected blood from non-human primates are infectious, could also occur in humans and is particularly important because it could partially explain the contrast between the inobservance of *P. falciparum* transfusional malaria cases in an area where an unexpected frequency of PCR positive for this parasite was registered. Finally, the third evidence comes from the confirmation of twenty-four randomly drawn *P. falciparum* positive blood samples retested by nested-PCR at the Laboratório de Pesquisa em Malária, Instituto Oswaldo Cruz, Fiocruz, according to the protocol of Zalis *et al.* [17]. Consequently, in view of the results reported by Maselli *et al.* [1] and the recent data quoted above, it seems evident that the dynamics of malaria in the Atlantic Forest is poorly known and needs further investigations, including studies relative to mosquito vectors and other determinants of the transmission, i.e., infectiveness and pathogenicity of the *Plasmodium* lineages that are circulating in the region, duration of infection in untreated individuals, duration of sporogony and gametocytogony, as discussed by Wernsdorfer [18].

Maselli *et al.* [1] have taken into account both the classical (bromeliad-malaria) and alternative patterns of the dynamics of malaria in the Atlantic Forest. However, the finding of *P. falciparum* DNA in blood donors in São Paulo suggests that the traditional dynamics of bromeliad-malaria is not the only one that can explain the presence of *Plasmodium* in areas of the Atlantic Forest in the state of São Paulo. Two major determinants of the bromeliad-malaria are the *Anopheles* (*Kerteszia*) mosquitoes and other *Plasmodium* species that do not belong to the subgenus *Laverania*. Cerruti *et al.* [19] addressed aspects of malaria in mountainous areas of the Espírito Santo state and hypothesized that, in the region, species of the subgenus *Nyssorhynchus* of *Anopheles* may be also involved in cycle of *Plasmodium* transmission. Thus, the results reported by Cerutti *et al.* [20] indicate, in agreement with the data of Maselli *et al.* [1], that a distinct dynamics of *Plasmodium* transmission could also occur in addition to the classical bromeliad-malaria.

Still considering the arguments against the presence of *P. falciparum* in Atlantic Forest by Mendrone *et al.* [20], it is worth noting that antibodies specific to the circum-sporozoite protein (CSP) of *P. falciparum* have been detected in simians from the Atlantic Forest in São Paulo state [21]. Moreover, Yamasaki *et al.* [22] surveyed monkeys from Atlantic Forest for *Plasmodium* infection, and concluded that the high proportion of positive sera against the CSP of *P. falciparum* was uncommon. It is interesting to point that Malafronte, a coauthor of the study byYamasaki *et al.*, also coauthored the paper by Mendrone *et al.* [20]. Additional support for the presence of *P. falciparum* in areas of Atlantic Forest, have been provided by Duarte *et al.* [23], who showed by PCR amplification that 1.4% of monkeys that tested positive for plasmodial infection had *P. falciparum* DNA. More important, the authors also surveyed monkeys both from urban areas and forest fragments in the vicinities of the municipality of São Paulo. Consequently, the arguments by Mendrone *et al.* [20] against the presence of *P. falciparum* are not supported by the data published in the literature.

The observation of asymptomatic individuals carrying *P. falciparum* DNA in higher prevalence among blood donors of a São Paulo Transfusion Center than in blood banks from the Amazon region is as intriguing as difficult to explain. However, if *P. falciparum* is circulating in areas of the Atlantic Forest in non-human primates and humans, it is plausible to assume that there are also mosquitoes infected with *P. falciparum*. The reasons for the lack of report of mosquitoes naturally-infected with *P. falciparum* are unknown; however, it is possible that the absence is, at least partially, explained by the methods employed to test mosquitoes, usually in pools [14, 24].

Cerutti *et al.* [19] employed immunofluorescence antibody test (IFAT) to observe a high percentage of human inhabitants of the Atlantic Forest of Espírito Santo state positive for IgM (13.5%) and IgG (13%) antibodies to *P. falciparum.* In addition, the authors reported nine individuals positive for *P. falciparum* by multiplex-PCR, and concluded that “the puzzling finding of *P. falciparum* DNA by multiplex PCR in asymptomatic individuals” and, more important, quoted that "the possibility of false positive results is remote as no other samples infected by *P. falciparum* were processed by PCR in the laboratory at the time of the study, thus ruling out the possibility of any cross-contamination". Consequently, the argument by Mendrone *et al.* [20] that "DNA supposed to be from *P. falciparum*… did not usually resist to a second amplification by another method" is not supported by data presented by team of his coauthor.

Mendrone *et al.* [20] also challenged the results of Maselli *et al.* [1] by arguing that in a PhD thesis associated to the article, an ELISA assay was utilized for testing the blood donors and the results were not included in the paper. This information is not incorrect. The thesis is authored by Aline Monteiro, one of the coauthors of the paper by Maselli *et al.* [1]. Although described as more sensitive than IFAT, the gold standard to estimate malarial antibody titers, the analytical sensitivity of ELISA-Malaria antibodies test kit from DiaMed (product 46460) was 40% with specificity of 98.3%, when tested in 923 malaria risk donors. According to Doderer *et al.* [25], the large number of non-concordant results between ELISA and IFAT impaired the performance of this DiaMed kit. Oh *et al.* [26] also reported that this test was insufficiently sensitive for blood screening for *P. vivax*. In addition to the described low performance of the DiaMed malarial kit, it has not been systematically tested regarding its sensitivity in low parasitaemia infection, it has not been approved by the Brazilian Agência de Vigilância Sanitária (Anvisa) and the kit production was interrupted. All these points were discussed in the thesis. Therefore, taking into account the negative features of the DiaMed kit, including its low sensitivity and inadequacy to detect subclinical infection, the results of the ELISA assays were considered inconsistent for publication.

Regarding mistakes in the references, the authors were aware of them and submitted an Erratum comment to the journal immediately after publication, which remains attached to the paper. This Erratum consists of a full list of corrections, including the correct number of autochthonous cases in São Paulo, and the fact that the Perandin *et al.* [27] citation replaces Gama *et al.* [28].

## Conclusions

The article by Maselli *et al.* [1] poses quite different challenges and has the merit of showing the presence of *P. falciparum* among asymptomatic blood donors and to emphasize that the current knowledge of malaria transmission in the Atlantic Forest domain is far too limited. The natural history of *P. falciparum* malaria in the Atlantic Forest is either poorly known or even unknown, including its vectors, hosts and reservoirs, both in spatial and temporal scales. There is an urgent need to improve the knowledge of the biotic and abiotic determinants as well as the natural history of the disease, before speculating and proposing any strict epidemiological profile for the dynamics of the *P. falciparum* in the biome. Most of the arguments by Mendrone *et al.* [20] regarding the discoveries of Maselli *et al.* [1] are based on the rationale that the results published are not supported by previous publications. On the contrary, it has been shown here that part of the previous published literature showed the same evidence. In agreeing with Thierry Maulnier, who says that “it would be unwise to believe that what never happened is impossible”, the reasoning by Mendrone et al. may be considered weak. Indeed, defending a stable and unchangeable pattern of bromeliad malaria, which was proposed in the 1940s and 1960s, to explain the malaria transmission in the Atlantic Forest now, is to deny any evolutionary processes that can drive the dynamics of the transmission of *Plasmodium* in a changing world.
